# Deciphering the Blood–Brain Barrier Paradox in Brain Metastasis Development and Therapy

**DOI:** 10.3390/cancers17020298

**Published:** 2025-01-17

**Authors:** Jens Jeshu Peters, Chubei Teng, Kang Peng, Xuejun Li

**Affiliations:** 1 Department of Neurosurgery, Xiangya Hospital, Central South University, Changsha 410008, China; 228119014@csu.edu.cn (J.J.P.); tengchubei@csu.edu.cn (C.T.); kangpeng01@csu.edu.cn (K.P.); 2 National Clinical Research Center for Geriatric Disorders, Xiangya Hospital, Central South University, Changsha 410008, China; 3 Hunan International Scientific and Technological Cooperation Base of Brain Tumor Research, Xiangya Hospital, Central South University, Changsha 410008, China; 4 Xiangya School of Medicine, Central South University, Changsha 410008, China

**Keywords:** blood–brain barrier, brain metastasis, metastasis therapy, focused ultrasound, nanoparticle

## Abstract

Gatekeeper or accomplice? That is the paradoxical role of the blood–brain barrier (BBB) in developing brain metastasis (BM). BM occurs when cancerous cells from primary cancer elsewhere in the body gain the ability to metastasize and invade the brain parenchyma despite the formidable defense of the BBB. These metastatic cells manipulate the BBB’s components, changing them from gatekeepers of the brain to accomplices that aid in their progression into the brain tissue. This dual role of the BBB—as both a protective system and a potential facilitator of metastatic cells—highlights its complexity. Even with metastasis therapy such as chemotherapy, BM usually recurs due to the BBB limiting the crossing of drugs via the efflux transporters; therefore, treatment efficacy is limited. The pathophysiology is also complex, and our understanding of the paradoxical interplay between the BBB components and metastatic cells still needs to be improved. However, advancements in clinical research are helping to bridge the knowledge gap, which is essential for developing effective metastasis therapy. By targeting the BBB neurovascular unit components such as the polarization of microglia, astrocytes, and pericytes, or by utilizing technological tools like focused ultrasound to transiently disrupt the BBB and therapeutic nanoparticles to improve drug delivery efficiency to BM tissue, we can better address this pathology. This narrative review delves into the latest literature to analyze the paradoxical role of the BBB components in the manifestation of BM and explores potential therapeutic avenues targeting the BBB–tumor cell interaction.

## Simple Summary

Although the blood–brain barrier (BBB) is an intricate, dynamic system that protects the brain and the central nervous system from negative influences, metastatic tumor cells from primary cancers such as lung, breast, melanoma, renal, colorectal, and gastrointestinal cancers can still bypass this protective barrier and develop cancerous lesions within the brain parenchyma, resulting in brain metastasis (BM). This suggests that the BBB plays a multifaceted role by not only acting as a protective system but also potentially facilitating the selective entry of these metastatic cells, contributing to the development and progression of BM. Therefore, understanding the duality of this role and how the BBB can be manipulated for therapeutic purposes is essential for developing targeted therapies that can effectively treat and prevent BM.

## 1. Introduction

Brain metastasis (BM) is a common and severe complication of solid tumor malignancies, affecting about one-third of patients and causing significant morbidity and mortality [1]. It is one of the most frequent types of Central Nervous System (CNS) tumors, with an incidence ranging from 70,000–400,000 new cases per annum and 10–40% of patients with primary tumors developing metastasis, outnumbering primary malignant brain tumors by a factor of 10 in the United States [2]. The majority of BM originate from primary cancers in the lung, breast, and melanoma. These cancers are particularly aggressive and have poor survival outcomes once they spread to the brain. However, other types of primary cancer, such as renal, colorectal, and gastrointestinal cancers, can also lead to BM, albeit less frequently [3]. The brain microenvironment (ME), which differs significantly from the ME of lesions outside the brain in terms of its cell types, anatomical structures, metabolic limitations, and immune environment, plays a vital role in the metastasis process. It exerts a unique and robust selective pressure on tumor cells, influencing both the metastatic process and treatment response [3]. Hence, understanding the intricate interplay between the constituents of the BBB and BM cells is of the utmost importance in devising innovative metastasis therapy for managing this pathology.

## 2. The Blood–Brain Barrier Overview

**Figure 1:**
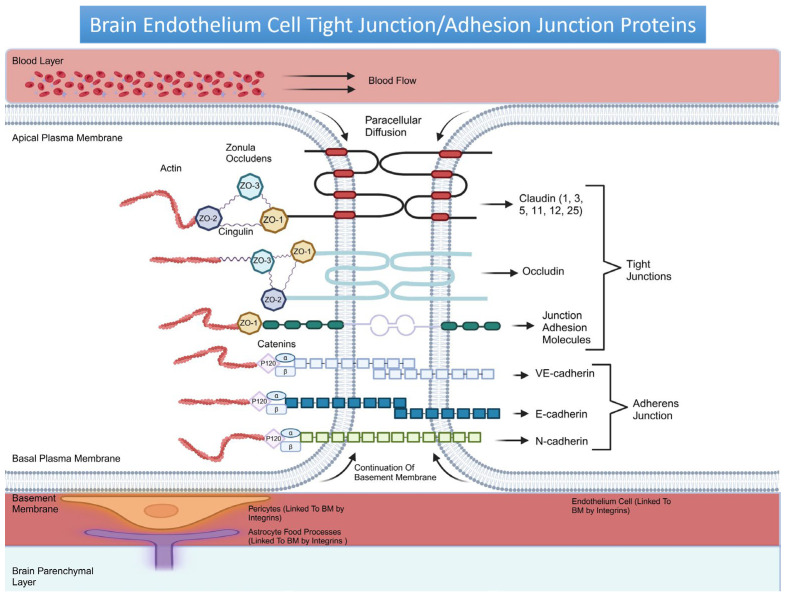
A schematic representation of BMEC TJ and AJ protein. It highlights the TJ protein claudins, characterized by four transmembrane domains, two external loops, one internal loop, a short cytoplasmic N-terminus, and a long cytoplasmic C-terminus. The extracellular loops are essential for maintaining TJ integrity and the epithelial barrier, with the shorter loop forming dimers between claudins on adjacent cell membranes [41]. The C-terminus of claudins anchors them to ZO-1-3 which facilitates scaffolding, cell-to-cell communication, cell polarity, and signal transduction [6,42,43]. Occludin has two extracellular loops flanking a single intracellular loop. Its N- and C-termini, facing the cytoplasm, interact directly with ZO proteins, while the extracellular loops from neighboring cells join to form the paracellular barrier [6,44]. JAMs have a single transmembrane domain, an IgG-like extracellular domain, and short cytoplasmic C- and N-termini. The C-terminus interacts with ZO-1 [6,45]. JAM-A and JAM-C are highly expressed in BMECs, while JAM-B’s expression is low [6,46]. Cingulin acts as a master regulator, interacting with other proteins and influencing critical cellular processes [47,48,49]. AJ proteins, VE-, E-, and N-cadherins are linked to the actin cytoskeleton via catenins [12,45,50].

The BBB is far more than just a static wall within the brain. It is a dynamic, intricate system with major components collaborating to support brain function, regulate the neural environment, and control molecular traffic. The BBB’s strength and selectivity stem from its intricate structural design. Brain Microvascular Endothelial Cells (BMECs), the main building blocks of this barrier, hold the key to its integrity by forming Tight Junctions (TJs) and Adherens Junctions (AJs) protein (see Figure 1). BMECs differ from those in other regions of the body, specifically expressing a lack of fenestrae, lower macropinocytosis and caveolar transcytosis levels, a reduced expression of the leukocyte adhesion molecule, and reduced integrin ligands and specific transport systems [4]. BMECs meticulously control the movement of substances in and out of the vasculature, with the Rho GTPase signaling pathway playing an essential role in maintaining BBB integrity by regulating BMEC TJ protein [5]. Additionally, the BBB protects the brain from toxins and pathogens, maintains ionic homeostasis, facilitates nutrient transport, removes waste products, and regulates neurotransmitter levels. It supports BMECs’ function through interactions with other components of the neurovascular unit (NVU), such as astrocytes and pericytes, which help maintain BBB integrity and function through signaling and structural support [4,5].

BMEC TJ includes transmembrane proteins like claudins, occludins, and Junction Adhesion Molecules (JAMs) anchored to the cell’s actin cytoskeleton through accessory scaffold proteins like Zonula Occludens (ZO) and cingulin, which helps fine-tune this selective permeability and ensures the structural and functional resilience of the BMECs [6]. Within the BBB, claudin-5, occludin, and ZO-1 are highly responsive markers of BBB permeability [7]. Recent findings also implicate claudin-1, -3, -5, -11, -12, and -25 as critical players in maintaining the BBB’s integrity, selective restriction of ion permeability, and functional interaction with occludin and JAMs [8,9]. Occludin has been shown to form heteropolymers with claudins, which might harbor dynamic channels. However, further investigation is needed to solidify this model [6], while the positive regulation of C/EBP-α by JAM-A enhances BBB strength via the claudin-5 protein [10].

AJs include cadherins, catenins, vinculin, and actinin, create an intercellular pathway between adjacent cells, and provide structural support [11]. VE-cadherin is pivotal in the development and preservation of the BMEC junctions, as it regulates the claudin gene expression by activating Wnt receptors, suppressing FoxO1 activity, and sequestering β-catenin through its linkage with the actin cytoskeleton [12]. The deletion of VE-cadherin compromises TJ integrity and disrupts the localization of ZO-1, claudin-1, and 4 [13]. E-cadherin strengthens cell–cell contacts and N-cadherin stabilizes FGFR and activates the MAPK/ERK and PI3K pathways for cell survival and migration [14].

The Basement Membrane (BME) further reinforces the BBB [15]. It regulates BBB permeability and integrity by interacting with extracellular matrix (ECM) proteins [16]. Components of the NVU anchor to the BME via integrins and dystroglycan receptors [17]. Extracellular α-dystroglycan links to BME proteins, while transmembrane β-dystroglycan connects α-dystroglycan to the actin cytoskeleton. Integrins, formed by α- and β-subunits, interact with vascular BME proteins and trigger signaling cascades. β1-integrins, expressed by BMECs, pericytes, and astrocytes, interact with collagen IV in the BME, influencing BBB integrity through the claudin-5 expression [17].

The NVU acts as the functional regulator of the BBB and consist of various cellular players. Astrocytes play a crucial role in enhancing TJ continuity within BMECs and regulating BBB permeability through Ca^2+^ signaling [18]. The structural integrity of astrocyte end-feet processes is significantly supported by Glial Fibrillary Acidic Protein (GFAP) and Vimentin [19]. However, under adverse conditions, reactive astrogliosis can be triggered, leading to increased GFAP expression. This response classifies astrocytes into two distinct phenotypes: A1 (neurotoxic) and A2 (neuroprotective) [20,21,22]. Pericytes facilitate the clearance of harmful substances from the brain and control blood flow by constricting or dilating blood vessels via the contractile proteins α-SMA, tropomyosin, and myosin [23,24,25,26]. Additionally, pericytes release signaling factors that guide the polarization of astrocytes and maintain BMEC integrity [27]. They also influence cellular processes and modulate the angiogenic factors VEGFR and NOTCH 3 [27]. The Shh signaling pathway in pericytes may mediate their impact on BMEC TJ protein production, thereby strengthening the BBB selective permeability [28].

Neurons play a limited role in the BBB, although the Spock1 gene has been shown to influence BBB development and maintenance [29]. Neuronal activity can also influence the expression of BBB efflux transporters and circadian genes in BMECs, dynamically regulating BBB function [30]. Recent research has indicated that neurons upregulate BBB protective functions by increasing the expression of claudin-5 and VE-cadherin TJ proteins through the secretion of glial-cell-line-derived neurotrophic factor (GDNF) [31]. Oligodendrocyte Precursor Cells (OPCs) are closely associated with neocortical gray matter capillaries, suggesting a role in stimulating angiogenesis. Through the HIF-activated secretion of Wnt and VEGF, OPCs may help adapt the local blood supply to the metabolic demands of OPC differentiation [32,33,34].

Although microglia are not a component of the NVU, they maintain CNS homeostasis by monitoring their environment and regulating neuronal activity [35]. Microglia exhibit M1 (pro-inflammatory) and M2 (anti-inflammatory) phenotypes [36]. In vitro co-culture experiments with mouse BMECs and resting microglia demonstrated the increased expression of essential TJ proteins [37,38,39]. However, recent research has shown evidence that microglia are not necessary for BBB maintenance, function, or gene expression in healthy brains [40].

## 3. Integrative Roles of the Glycocalyx and Biochemical Barriers in BMECs

### 3.1. Glycocalyx Layer

The glycocalyx layer serves as both a physical and charged barrier on BMECs. It repels interactions with circulating molecules, preventing the infiltration of plasma components into the endothelial domain, and regulates interactions between plasma and BMECs [51]. Additionally, it plays a pivotal role in drug delivery across the BBB. By restricting the passage of large-molecule drugs and biologics, it acts as a crucial regulatory mechanism for their transport [52]. The negatively charged nature of the glycocalyx enables the selective binding of positively charged entities, with electrostatic interactions influencing drug adhesion and movement across this layer [51]. The glycocalyx also expresses mechanosensory characteristics, participating in inflammatory and anticoagulation processes, and regulating the BBB ME. Its diverse glycosaminoglycan (GAG) sugar chains not only provide binding sites for various molecules, altering the local concentration of Fibroblast Growth Factor Receptor (FGFR) and downstream effects on gene transcription and development, but also help stabilize and integrate the glycocalyx, enhancing its barrier properties [51,53].

**Figure 2:**
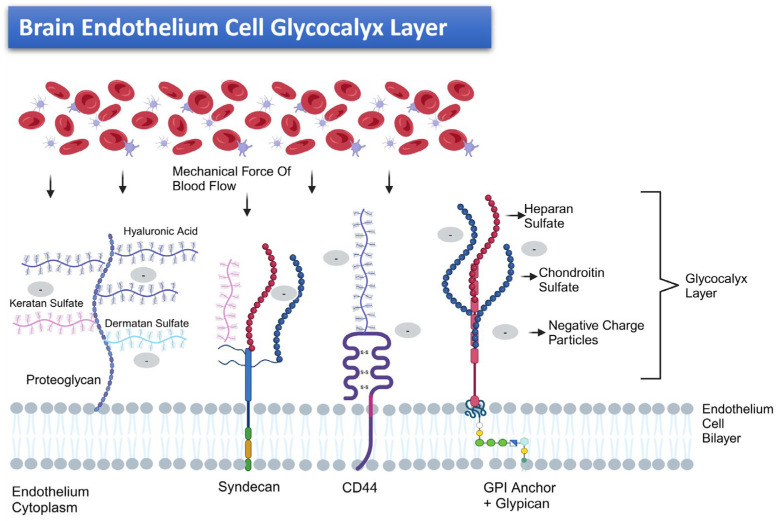
An illustration of the BMECs’ glycocalyx layer, a complex structure of proteoglycans (PGs) and anionic GAGs. The structure features various GAG types—notably, heparan sulfate (HS), which dominates the side chains (50–90%), along with chondroitin sulfate (CS), keratan sulfate, dermatan sulfate, and hyaluronic acid [54]. The glycocalyx’s core proteins anchor to the endothelial membrane through diverse mechanisms: CD44 association, syndecans, and glypicans. This elaborate coating shields the luminal endothelial surface, promoting stability in the BBB [4,54]. Syndecans are prominently expressed on the vascular endothelium and uniquely bind multiple GAGs. They exhibit remarkable responsiveness to the mechanical forces of blood flow [51,55]. The negatively charged essence of the glycocalyx stems from the abundant sulfation of GAGs and the plethora of sulfate residues and hyaluronic acid integrated into its sugar chains, providing it with a robust electrostatic barrier [51,55].

Disruptions of the glycocalyx can lead to the increased permeability of the BBB, allowing harmful substances to enter the brain, contributing to conditions like neuroinflammation and neurodegenerative diseases [52]. Moreover, damage to the glycocalyx can result in increased oxidative stress within the brain, which may exacerbate the progression of diseases such as Alzheimer’s and Parkinson’s [52]. During ischemic strokes, the degradation of the glycocalyx exacerbates endothelial dysfunction and increases BBB permeability, leading to further brain damage and inflammation. These interactions can impact the expression of TJ proteins and transporters involved in drug transport, potentially altering the permeability of the BBB [52]. Additionally, when the glycocalyx is compromised, there is an increase in the adhesion of leukocytes to the endothelial cells, which can lead to chronic inflammation [52]. An illustration of the BMECs’ glycocalyx layer is provided in Figure 2.

### 3.2. Biochemical Barrier

**Figure 3:**
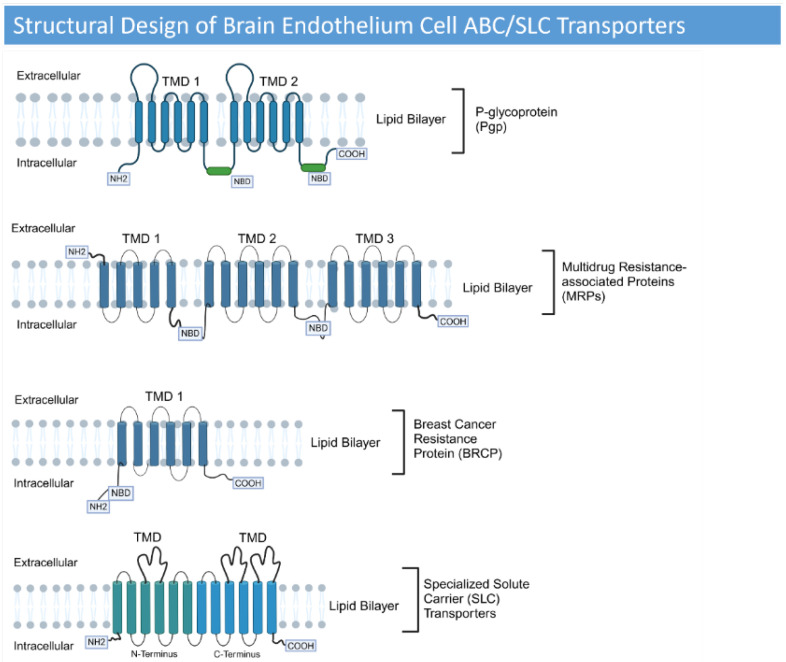
A schematic diagram of the ABC and SLC transporters. ABC transporter P-gp structurally has two homologous moieties containing six transmembrane domain (TMD) helices and a nucleotide-binding domain (NBD) fueled by ATP. This configuration is paramount for P-gp’s role in drug resistance. The MRPs share a similar architecture of transmembrane helices embedded within a membrane-spanning domain (MSD) and an intracellular nucleotide-binding domain for ATP interaction. MRP1, MRP2, MRP3, MRP6, and MRP7 possess an additional NH2-proximal MSD, hinting at potential functional specialization [56]. BCRP possesses a more asymmetric structure, consisting of a solitary ATP-binding domain and membrane-spanning region [56]. SLC transporters harness electrochemical gradients rather than ATP; therefore, there is no NBD. They employ facilitated diffusion, ion-coupled transport, and exchange mechanisms to achieve this feat [59].

BMECs have transporters, including ATP-binding cassette (ABC) and Solute Carriers (SLCs). P-glycoprotein (P-gp), Multidrug Resistance-Associated Proteins (MRPs, ABCC), and Breast Cancer Resistance Protein (BRCP, ABCG2) are prominent ABC transporters that expel toxins and drugs from the brain [30]. The MRP family of transporters also demonstrates a remarkable capacity to efflux a vast spectrum of compounds, both xenobiotic and endogenous [56]. The SLC transporters play a vital role in mediating the exchange of substances across the BBB [57]. SLC transporters are crucial for acetylcholine production, oxidative stress mitigation, carnitine handling, and neurodegenerative disease prevention [58]. The structural design of ABC and SLC transporters is illustrated in Figure 3.

In addition to transporters, drug-metabolizing enzymes, such as cytochrome P450 enzymes (CYP1B1, CYP2U1, and CYP3A4), contribute significantly to the formation of the biochemical barrier by metabolizing drugs and fatty acids [6]. For example, CYP2U1 metabolizes fatty acids like arachidonic acid, while CYP1B1 is involved in the metabolism of endogenous compounds in the CNS. CYP3A4 was also shown to be functionally expressed in the drug resistance of epileptic BBB and oxidizes a large group of xenobiotics, including antiepileptic drugs [6]. These enzymatic activities play a crucial role in maintaining the protective functions of the BBB by detoxifying harmful substances and regulating their passage across the barrier. This activity helps preserve the homeostasis of the brain ME, shielding it from potentially harmful compounds [6].

However, there are pathological implications when the biochemical barrier is disrupted. Such disruptions can lead to increased BBB permeability, allowing harmful substances to enter the brain and contribute to the progression of neurodegenerative diseases like Alzheimer’s and Parkinson’s [6]. For instance, an animal model of Alzheimer’s disease revealed that impairment in ABC transporters leads to the accumulation of amyloid β-peptide in the brain. Additionally, disruptions can provoke inflammation, exacerbating conditions such as multiple sclerosis and oxidative stress, further contributing to the progression of neurodegenerative diseases [6].

## 4. Immunosurveillance: Guardians or Accomplices in Brain Metastasis

The brain’s immune surveillance system relies heavily on resident microglia and astrocytes as its first line of defense. These cells are complemented by peripheral immune cells, such as T-cells and Natural Killer (NK) cells; however, the focus would primarily be on microglia and astrocytes and their role in suppressing and promoting BM development and their potential therapeutic modalities.

### 4.1. Microglia: Chief Immune Sentinels or Paradoxical Protectors

Microglia play a pivotal role in the brain’s immunosurveillance network by maintaining a dynamic communication loop with neurons in their resting state. This exchange of environmental cues informs microglia about the brain’s health status, making them a critical defense line against metastatic invasions [60]. In response to specific stimuli, microglia differentiate into distinct phenotypes: M1, which releases pro-inflammatory cytokines (IFN-γ, TNF-α, and IL-1β), chemokines (CCL2, CXCL9, and CXCL10), protein hydrolases, iNOS, and reactive oxygen species, creating a cytotoxic environment hostile to tumor cells [61]. M2 express anti-inflammatory characteristics through signaling mechanisms via IL-4, FCc, and IL-10 receptors [36,39]. The activation of the IL-4 receptor triggers phagocytic mechanisms linked with tissue repair, promoting anti-inflammatory microglial polarization [39,62]. Interestingly, the M2 phenotype can indirectly promote tumor survival and proliferation by secreting immunosuppressive factors such as Transforming Growth Factor-β (TGF-β), IL-10, and CCL20 [63].

Preclinical observations suggest that microglia are active during the extravasation stage of BM development [64]. Microglia usually transition into tumor-associated macrophages (TAM-MGs) within the metastatic ME and play a pivotal role in shaping the course of BM, initially contributing to tumor cell elimination through host defense mechanisms. However, cancer cells eventually employ various strategies to subvert their function, tipping the balance toward tumor progression [65]. The transition mechanisms of microglia into TAM-MGs are still elusive. However, emerging research indicates that the ’instruction’ and phenotypic shift occurs within the early stages of BM development. Transcriptional analysis suggests minimal variation in the genes expressed by TAMs across different tumor sizes. This points to a potentially stable set of instructions driving TAM behavior throughout tumor progression, although further studies are essential in order to fully elucidate these mechanisms [63].

TAMs are abundant within the tumor ME, constituting up to 50% of live cells in glioblastoma and a similar presence in BM [66,67]. Within these metastatic lesions, TAMs directly infiltrate the tumor tissue [68]. In vivo studies demonstrate that TAMs facilitate the tumor cell invasion of brain tissue by reprogramming immune cells, fueling tumor growth by suppressing the immune response, reshaping the ECM, and promoting angiogenesis [69]. TAMs also release nitric oxide, which functionally dilates blood vessels, draining essential amino acids crucial for cytotoxic T-cells and effectively sabotaging their anti-tumor activity. Furthermore, TAMs disrupt IL-2 signaling, further hindering the immune response. The microglia expression of Neurotrophin (NT)-3 plays a crucial role in regulating immune cell activation and promoting neurogenesis. Unfortunately, cancer cells have been known to exploit this mechanism by taking advantage of NT-3 to encourage the formation of BM [70,71].

As metastatic cells breach the BBB during extravasation, microglia will mount an initial response through a limited upregulation of IL-6, a cytokine with a paradoxical effect. While initially intended as a defensive measure, IL-6 surges create an immunosuppressive environment, dampening the activity of T-cells and potentially hindering the effectiveness of immune checkpoint blockade therapies [72]. Investigations of IL-6, microglia, and Non-Small-Cell Lung Cancer (NSCLC) have revealed this correlation. Patients with higher serum IL-6 levels exhibited a greater susceptibility to BM, while data from the cancer genome atlas indicated that individuals with lower IL-6 levels had improved overall survival rates [73]. Beyond IL-6, microglia also release C-X-C motif chemokines 5 and 8 (CXCL5 and CXCL8) in response to BM. These “siren molecules” act as deceptive signals, luring immunosuppressive neutrophils, ultimately weakening the immune response and favoring tumor progression [65].

Microglia’s role in BM extends beyond a one-size-fits-all strategy. In melanoma BM (MBM), these tumor cells secrete amyloid-β, effectively removing microglia’s phagocytic abilities and hindering their ability to clear tumor debris [74]. Nicotine has also been demonstrated to prompt the switch to the M2 phenotype via the nAch receptor-STAT3 pathway and the upregulation in SIRPα expression. This transition results in the elevated production of IGF-1 and CCL20, promoting the growth of metastatic tumors [75]. Research also revealed that microglial exosomes play a pivotal role in Breast Cancer Brain Metastasis (BCBM), as these microglial-derived vesicles seem to influence metastatic progression. Notably, exosomes containing miRNA-503, secreted by microglia due to the XIST deletion in BCBM cells, contribute to the phenotypic switch from M1 to M2. This phenomenon appears to be mirrored in lung cancer BM (LCBM), where endogenous Dkk-1 released by BMECs elicits a similar microglial polarization [76].

### 4.2. Microglia Polarization as a Potential Therapeutic Target

Targeting the polarization of microglia has shown positive therapeutic potential. The receptor tyrosine kinase VEGFR2 is vital in guiding microglia toward the M2 phenotype. However, the inhibition of VEGFR2 kinase with a Flk-1 inhibitor disrupts this polarization process, highlighting a crucial pathway in microglia modulation [39,77]. The signaling pathway, IL-6/JAK2/STAT3, is also involved in microglial polarization and has been successfully targeted using Tocilizumab. This monoclonal antibody neutralizes IL6R and fedratinib, a JAK2 inhibitor that inhibits the polarization of M2 microglia. These treatments have been shown to reduce the incidence of BM in high-risk NSCLC patients [73].

In the context of MBM, the phyto-glyceroglycolipid component 1,2-di-O-α-linolenoyl-3-O-β-galactopyranosyl-sn-glycerol (dLGG) has demonstrated the ability to convert M2 microglia to M1. Additionally, dLGG influences the secretion of 9,10-EpOMEs + 12,13-EpOMEs, which can help prevent invasion and facilitate the M2 to M1 transformation [78]. The pharmacological inhibition of anti-inflammatory macrophages through either Colony Stimulating Factor 1 Receptor (CSF-1R) or STAT6 pathways has significantly the decreased tumor burden in BCBM. This further proves that polarization from the M2 to M1 phenotype may be an effective therapeutic strategy for treating BM [79].

### 4.3. Astrocytes: Allies in Immune Defense or Paradoxical Partners

In the early stages of BM invasion, astrocytes mount a formidable immune defense against encroaching tumor cells, serving as vital allies in protecting the brain and CNS [80]. Astrocytes defend the brain by synthesizing plasmin and FasL, which eliminate cancer cells. Plasmin prevents tumor cell adhesion by cleaving L1CAM and facilitating the release of FasL. However, cancer cells can inhibit plasmin production by expressing anti-PA serpins, allowing them to establish themselves in the brain [81,82]. Astrocytes are abundant in the CNS and may play a role in intercellular communication. Studies suggest they release extracellular vesicles (EVs) in response to IL-1β, which alters the cytokine dynamics and facilitates leukocyte infiltration into the brain [83,84].

Astrocytes shift their role to become allies of the invading tumors as the tumor’s potential to spread increases. This shift is not well-understood but involves interactions with neighboring cells and signals emitted by cancer cells. In their pro-tumor state, astrocytes promote tumor growth, spread, and protection from the immune system [85]. An investigation into miR-146a-5p, a microRNA found in high concentrations in EVs from MBM, found that it is transmitted to astrocytes, inhibiting the protein NUMB in the Notch signaling pathway. This activates the cytokines IL-6, IL-8, MCP-1, and CXCL1, which promote tumor growth. The knockdown of miR-146a-5p resulted in a significant decrease in BM and a reduction in the levels of these cytokines in astrocytes. The compound deserpidine was identified as a functional inhibitor of miR-146a-5p, demonstrating efficacy both in vitro and in vivo as a potential adjuvant treatment for targeted therapy [86].

Astrocytes significantly influence the invasion of metastatic cells across the BBB by secreting C-C motif chemokine ligand 2 (CCL2), a potent attractant for metastatic cells. When CCL2 binds to the C-C chemokine receptor type 2 (CCR2) on the tumor cells, it enhances mobility and directs their infiltration into brain tissue [87]. Astrocytes also create a type I interferon (IFN) ME, which activates the production of CCL2, increasing the recruitment of monocytic myeloid cells, which facilitates the carcinogenesis and metastasis processes [88]. However, it was demonstrated that blocking CCR2 genetically or using the drugs cenicriviroc (CVC) and 15a can significantly reduce the spread of BCBM and MBM [88].

Metastatic cancer cells prompt astrocytes to increase the Jagged1 expression, which activates NOTCH signaling in cancer stem-like cells, leading to the production of HES5, a protein vital for cancer stem cell self-renewal and proliferation [89]. Moreover, metastatic tumors form gap junctions with astrocytes through the combined action of connexin 43 (CX43) and protocadherin 7 on cancer cells, allowing for the exchange of signaling molecules. Cancer cells further secrete cyclic GMP-AMP, which activates the STING pathway, producing the inflammatory cytokines IFN-α and TNF-α in astrocytes. These cytokines trigger the activation of STAT1 and NF-κB pathways, promoting metastatic growth and resistance to chemotherapy. Astrocytes are also calcium sequesters, which prevent chemotherapy-induced apoptosis in cancer cells, further contributing to therapy resistance [90]. Genetic evidence suggests that decreasing the gap junction signaling may reduce the development of BM. Animal models have demonstrated that Meclofenamate, a known Cx43 gap junction gating inhibitor, can produce this effect. Tonabersat, a benzopyran derivative that binds to astrocytes, also inhibits gap-junction-mediated processes. Moreover, both Meclofenamate and Tonabersat have been found to lower the release of IFN-α and TNF-α in astrocyte cancer cell co-cultures [90].

Reactive astrocytes with STAT3 create a favorable environment around the metastatic lesion, facilitating tumor progression. STAT3 activity is critical in the later stages of BM and correlates with poor clinical outcomes [91]. Moreover, it was found that reactive astrocytes expressing phosphorylated STAT3 (pSTAT3) promote the invasive growth of BM. Inhibiting STAT3 in these astrocytes leads to a delay in their development. These astrocytes additionally secrete Chitinase 3-like-1 (CHI3L1), inducing cancer cell invasion into the brain. STAT3 and CHI3L1 could be potential therapeutic targets for treating highly invasive BM [92].

In MBM, astrocytes contribute to the invasion process by influencing cellular navigation and communication. In particular, CCR4 and its ligand CCL17 secreted by astrocytes are a potent axis in the metastatic process. Acting like a homing beacon, CCL17 attracts CCR4-positive melanoma cells, and their interaction leads to the expression of MMP13, enhancing metastatic cell invasiveness [93]. MBM cells also impact astrocyte behavior by “reprogramming” astrocytes to produce IL-23. This cytokine increases MBM cell invasion through the BBB and promotes the activity of MMP2, which, in turn, promotes tumor growth and angiogenesis within the brain [94]. As fatty acid producers, astrocytes can inadvertently fuel PPAR-gamma activity in cancer cells. This pathway, which is linked to metabolism, inflammation, and excessive cell growth, is ultimately co-opted by cancer cells, enabling their metastatic goals [95,96].

BCBM research revealed that the transcription factor TGL1 is essential in activating breast cancer stem cells (BCSCs) [97]. This study provides compelling evidence that TGL1 strengthens the “stemness” of BCSCs by increasing the expression of genes such as CD44, OCT4, and Nanog, which are essential for stem cell self-renewal and pluripotency. Furthermore, TGL1-positive BCSCs trigger reactive astrogliosis, potentially indicating a feedback loop, where the TGL1 activation in BCSCs induces reactive astrogliosis and creates a favorable environment for metastatic progression.

Insights into Smal Cell Lung Cancer (SCLC) BM also revealed that metastatic cells can potentially co-opt developmental cues. Qu et al. highlighted the role of reelin, a molecule crucial for brain development, suggesting that SCLC cells exploit reelin’s natural function by secreting it to manipulate astrocytes. Reelin, typically involved in neuronal migration during development, appears to “trick” astrocytes into misinterpreting the signal. This miscommunication leads astrocytes to provide “survival factors” that support SCLC growth within the brain ME. Targeting the reelin signaling pathway and SERPINE1 inhibitors has shown promising therapeutic viability [98].

## 5. Pericytes: Regulators or Facilitators in Brain Metastasis

Pericytes modulate BBB integrity through their close association with BMECs, facilitating dynamic crosstalk that reinforces BBB function. This interaction promotes the expression of TJ proteins, safeguarding the brain from the invasive influence of cancerous cells [27,99,100]. Further highlighting their protective function, Fujimoto et al. demonstrated the ability of pericytes to suppress LCBM cells in an in vivo model. Their work suggests that pericytes, through complex interactions with neighboring cells like astrocytes, may secrete factors that bolster BBB function and impede the progression of metastatic cells. Identifying these specific pericyte-derived factors remains a crucial area of investigation [101,102].

The interactions between pericytes and cancer cells can lead to changes in the gene expression profiles of some pericytes. A laboratory model investigating pericytes, BMECs, and LCBM cells together found that pericytes considerably hindered the growth of cancer cells. Upon analyzing specific batches, it was observed that two genes, Wwtr1 and Acin1, were less active and enrichment analyses revealed the inhibition of apoptotic processes in fibroblasts [103]. However, it is equally important to acknowledge the potential pro-tumorigenic functions of pericytes.

There is a dynamic relationship between pericytes and BMECs during vascular remodeling. When pericytes detach from BMECs, it can trigger the sprouting and growth of the endothelium. This process is governed by signaling pathways that involve Angiopoietin-1/2 and Tie2 (Ang/Tie2), TGF-β, and Platelet-Derived Growth Factor-B (PDGFB) and its receptor PDGFR-β [104]. Within the tumor ME, cancer cells frequently overproduce PDGF-BB, resulting in sustained high levels of this growth factor. This overproduction can decrease the expression of α1β1 integrin adhesion receptors in pericytes by downregulating PDGFβR. This reduction can cause the detachment of pericytes from the ECM of blood vessel walls. Such detachment can then promote tumor angiogenesis by allowing tumor cells to attract pericytes and increase vascular permeability [104,105,106]. Moreover, research indicates that PDGF-BB can induce the pericyte-to-fibroblast transition (PFT). This transition has been shown to promote metastasis development [107].

The breakdown of the BME is necessary for angiogenesis and metastasis progression, and pericytes are actively involved in this process by secreting MMPs [100]. VEGF and TGF-β are the key molecular players in this scenario, with degenerating pericytes releasing elevated VEGF, further solidifying the link between angiogenesis and tumorigenesis [100]. Interestingly, the TGF-β pathway demonstrates duality, acting as a tumor suppressor in early tumorigenesis but promoting metastasis in late-stage cancer. NOTCH3 also fuels angiogenesis by promoting EMT, allowing tumor cells to gain stem-cell-like properties and divide and disseminate uncontrollably [100,108,109].

### Treatment Potential of Targeting Pericytes

Regarding the therapeutic potential of pericytes, they secrete insulin-like growth factor 2 (IGF2), which has been found to stimulate the growth of BCBM cells. Interestingly, this substance appears not to affect MBM cells. However, by inhibiting IGF2 signaling via genetic manipulation and drug therapy using picropodophyllin (PPP), the growth-stimulating effect of pericytes on BCBM cells can be successfully counteracted [110].

Further evidence revealed a role for CD44+ lung cancer stem cells in promoting BM. These cells can transform into vascular pericytes with remarkable migratory abilities through a receptor known as GPR124. This receptor triggers the initiation of the Wnt7-β-catenin signaling pathway, allowing these transformed cells, now called ‘CD-pericytes,’ to enter blood vessels, survive in circulation, travel to the brain, and then revert to their original stem cell state to form tumors. Targeting CD-pericytes, GPR124, or the Wnt7-β-catenin pathway could be a potential therapeutic approach to disrupting this metastatic process [111].

CD276, a member of the B7 family, has been identified as a potential target for cancer immunotherapy due to its role in promoting cancer cell aggressiveness and facilitating angiogenesis within tumors. It is overexpressed in BMECs and pericytes; thus, the use of a CD276-blocking antibody has shown a significant increase in survival rates and a substantial reduction in tumor size [112].

## 6. Neurons and Oligodendrocytes: Indirect Contributors in Brain Metastasis

While research on the role of oligodendrocytes in BM remains limited, there is evidence showing they may contribute to the metastatic process indirectly by producing chondroitin sulfate proteoglycan, hindering the regrowth of nerve fibers, potentially restraining healing and indirectly aiding tumor expansion [113,114]. Additionally, inflammation triggered by invading tumors, alongside reactive astrocytes and microglia, functionally compromises oligodendrocytes, disrupting myelin, leading to further inflammation, and potentially contributing to tumor progression and treatment resistance [21,115,116]. Regarding therapy, 68Ga-PSMA PET/CT imaging and single-cell RNA sequencing revealed that prostate-specific membrane antigen (PSMA) protein is notably present in oligodendrocytes found in LCBM. This increased presence of PSMA could be linked to stress in the endoplasmic reticulum and the response to unfolded proteins, which may be regulated by ATF3 and NR4A1. This discovery could pave the way for the use of PSMA radioligand therapy in BM therapy [117].

In the case of neurons, the neuron–BM relationship remains elusive, although evidence indicates that this relationship induces neuro-transmitters and synaptic signaling mediators. BCBM cells have been shown to acquire GABAergic responsiveness and contribute to the development of BM upon the exposure to neurons. Furthermore, the neuron-mediated reelin resurgence in tumor cells has been found to facilitate early CNS adaptation in vitro [115,118]. Observations revealed decreased GABA levels in BCBM cells, which are associated with a poor prognosis. Additionally, there was an increased expression of reelin, which aids in the integration of tumor cells within the brain parenchyma [118]. Comparable observations have been documented in SCLC BM research, where these cells exhibit ‘neuronal mimicry’, undergoing dynamic transformation to resemble neurons as the tumor progresses [119].

The neurotransmitter glutamate may also contribute to the growth of MBM, but the exact mechanism remains unclear, although evidence suggests the cannabinoid CB1 receptor (CB1R) may contribute. CB1Rs typically reduce the amount of glutamate released by nerve cells. Further analysis found that blocking the selective blockade of glutamatergic NMDA receptors (but not AMPA or metabotropic receptors) reduced cell growth. Furthermore, in mice lacking CB1Rs, the tumors grew more extensively in the brain. These findings suggest that CB1Rs in nerve cells may be crucial in controlling metastatic growth, offering a potential new therapeutic avenue [120].

Receptor tyrosine kinase Ret proto-oncogene (RET), a signaling receptor for GDNF, has also been implicated in metastasis development. Evidence has shown that RET is overexpressed in BCBM. This overexpression highlights a potential therapeutic strategy using RET inhibitors for patients with BCBM [121].

## 7. Basement Membrane: An Invasive Platform in Brain Metastasis

Studies indicate that vascular co-option plays a crucial role in tumor growth. The protein β1 integrin is implicated in the initiation and progression of various cancers. β1 integrin facilitates the attachment of cancer cells to the ECM and BMECs, by binding to specific ligands such as fibronectin, collagen, and laminin [122]. This adhesion is essential for cancer cells in order to establish a foothold in brain tissues and form metastases. Inhibiting β1 integrin disrupts these adhesion processes, potentially preventing the establishment and development of BM [122,123].

The invasion of lung cancer cells into the brain results in the development of the blood–tumor barrier (BTB). To better understand the alterations in the BTB and the brain area surrounding the tumor (BAT), a team of researchers devised a model to observe these changes over time in NSCLC BM. They observed a decrease in collagen IV in the BTB of experimental NSCLC BM, but an increase in laminin-α2 in the BAT. However, the expression of laminin-α2 in the BTB was variable and resembled the BBB after six weeks of colonization. In specimens of human BM, there was a notable difference in the expression of collagen IV between the BTB and BAT, characterized by evidence of perivascular edema and a discontinuous expression pattern. There was a loss of expression in LAMA2 in the BTB compared to the BAT, which also showed a discontinuous expression pattern. These findings suggest that changes in the BME may help predict BBB permeability [124].

The stiffness of the tissue ME has been identified as a critical factor in the spread of cancer cells, challenging the previous belief that pore size was the primary determinant. Evidence has demonstrated that the rigidity of the ECM surrounding tissues significantly influences metastatic progression. By reducing the expression of the pro-adhesive protein netrin-4, it is possible to modulate the ECM’s stiffness, rendering it more pliable even with larger pores. This discovery refutes the conventional theory that larger pores facilitate the easier transmigration of cancer cells. Instead, it reveals that softening the ECM, despite an increased pore size, can hinder the spread of cancer cells. These findings open new avenues for exploring netrin-4 as a potential therapeutic target to address the challenge of cancer metastasis [125].

## 8. Endothelial Cells: Gatekeepers or Collaborators in Brain Metastasis

Metastatic cells utilize two methods to cross the BBB—paracellular through endothelial TJs, and transcellular—with evidence showing that BCBM cells invade transcellularly from the apical to the basolateral of BMECs, a process not influenced by N-cadherin [126,127]. The CD31 protein concentration in BMECs was reported to be higher in the BTB and BAT than the BBB, particularly during the middle and late stages of metastasis. The analysis of NSCLC BM showed that the blood vessels in the BTB were thicker, more distended, and had greater CD31 levels when compared to the BAT, indicating changes in BMECs’ TJ and increased vascular permeability, which can facilitate the spread of metastatic cells [124]. Cancer cells attach to BMECs during the extravasation stage of metastasis through various adhesion receptors, including integrins, CD44, and MUC1, which significantly stabilize this adhesion and potentially utilize the glycocalyx layer. This stabilization leads to further interactions with different types of cells present in the bloodstream, ultimately contributing to the process of extravasation [128,129]. In vitro models demonstrated that tumor cells exhibiting a strong adhesive ability towards BMECs, expressing elevated levels of MUC1, VCAM1, and VLA-4, and targeting these adhesion molecules might lead to possible treatment strategies [130].

The glycocalyx has different effects on normal BMECs and tumor cells. It inhibits tumor cell adhesion and migration across a healthy endothelium while promoting metastasis by the tumor cell glycocalyx [131]. Disturbed flow conditions can compromise the glycocalyx, leading to an increased attachment of circulating tumor cells (CTCs) to the endothelium, an essential step in tumor formation. It was found that disturbed flow and a 50% reduction in specific glycocalyx components led to a doubling in CTC attachment to the BMECs [132]. Additionally, VEGF appears to degrade the glycocalyx in BMECs while enhancing it in malignant BCBM cells. This dual effect facilitates the attachment and transmigration of BCBM cells across the BBB [133].

Modulating HS levels in the BBB and cancer cells may serve as a potential therapeutic strategy. Research indicates that manipulating HS levels on both cancer cells and the BBB can regulate cancer cell attachment, influencing their transmigration across the BBB. Utilizing specific substances like orosomucoid, S1P, and GM6001 to reduce HS levels in cancer cells while increasing them on the BBB has been shown to decrease cancer cell adhesion, thereby enhancing the BBB’s protective function. These findings suggest that targeting HS levels could be an effective approach to preventing the spread of BCBM [134].

### 8.1. Efflux Transporters: The Silent Accomplices in Brain Metastasis

Efflux transporters significantly impact BM, especially from a drug delivery standpoint, as they are responsible for the expulsion of therapeutic drugs. These transporters, such as P-gp and MRPs, actively pump out chemotherapeutic agents from the brain, reducing the intracerebral concentration of these drugs and thereby limiting their efficacy [135]. This results in reduced intracellular drug accumulation, rendering the treatments less effective. In cancer cells, the overexpression of these transporters can lead to multidrug resistance, where tumors become resistant to a range of chemotherapeutic agents. This mechanism is a major challenge in treating BM, as it necessitates the development of strategies to bypass or inhibit these efflux transporters to improve drug delivery to the brain [135].

**Figure 4:**
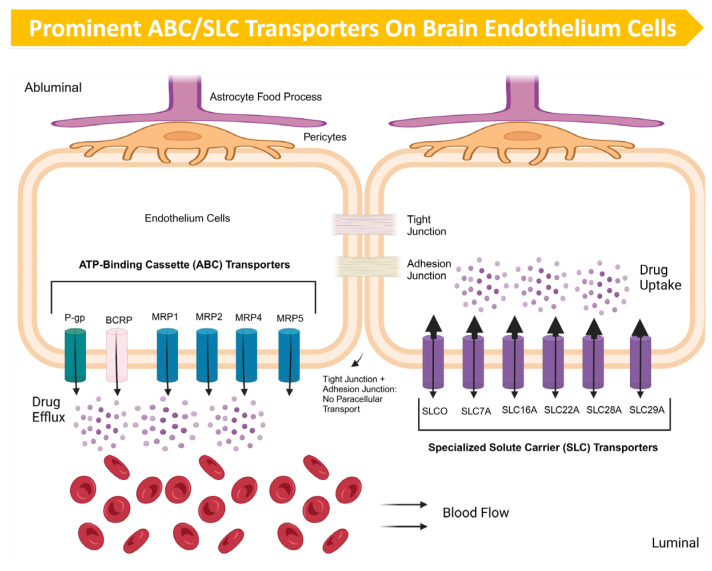
An illustration of the prominent ABC transporters on BMEC, including P-gp, MRPs, and BRCP and SLC transporters. Evidence suggests that Pgp and BCRP are highly expressed on the BMEC’s luminal membrane and may strategically collaborate in their protective function and share transport duties [6]. The SLC families, including SLCO, SLC7A, SLC16A, SLC22A, SLC28A, and SLC29A, are strategically expressed on both sides of the BMEC; they orchestrate bidirectional substrate movement and intriguingly favor drug uptake over efflux [58,59].

Despite the increased permeability of the BTB observed surrounding tumors, efflux transporters can maintain functional integrity, representing a significant obstacle in drug delivery (see Figure 4). The expression levels of P-gp and ABCG2 were investigated in lung and BCBM patients. Over 80% of the specimens showed a disruption of the BBB in terms of efflux transporter function, potentially explaining why some BM cases respond favorably to chemotherapy. However, a subset still had an intact BBB with functional efflux transporters, which could explain the observed challenges in treating some BM cases [136,137]. Evidence has also revealed elevated levels of P-gp and BCRP on both the luminal membranes of endothelial cells and the plasma membranes of tumor cells within the BTB environment [138].

Efflux transporter modulation in the BBB also varies according to the tumor type. Studies in adenocarcinoma BM indicate a stability in P-gp and BCRP levels, increased MRP1 and MRP3, and decreased MRP4 expression. Conversely, in glioblastoma, there is a reduced P-gp and BCRP expression, elevated MRP3 and MRP5, and unchanged BCRP [135]. With regard to SLC transporters, the precise function within BM development remains a field of active investigation, with preliminary reports hinting at their potential involvement in drug resistance [139].

### 8.2. Structural Protein Role in Brain Metastasis

TJ and AJ proteins are critical components of the BBB, forming a significant barrier to invasive metastatic cells. These structural proteins form a selectively permeable barrier which regulates the passage of cells and molecules between the bloodstream and the brain. These structural proteins maintain the integrity of the BBB by sealing the gaps between BMECs, thereby protecting the CNS from harmful substances and pathogens [12].

However, despite their significant role in forming a barrier to metastatic cells, disruptions in these structural proteins can profoundly affect the development of BM. Studies in mice revealed that lower levels of claudin-1 lead to MBM development. Conversely, boosting claudin-1 expression impedes these cells from spreading to the brain, suggesting that claudin-1 levels may be a determining factor in developing MBM [140]. MBM has also been found to disrupt the integrity of TJ in the brain by affecting the expression of claudin-5, ZO-1, occludin, and VE-cadherin, which can lead to a breakdown in TEER, boosting the tumor cell invasion potential [93,141,142].

Decreased claudin-5 has been linked to peritumoral edema, a common feature observed in BM. Moreover, claudin-5 plays a crucial role in various processes in vascular BMECs, such as motility, matrix adhesion, and angiogenic potential, which may impact cancer cell interactions with the blood vessels [143]. An observable trend in the expression of claudin-5 has emerged, revealing that, as cancer progresses, its expression increases, but its pattern changes from a linear thread-like form to a clumped and thickened one. This alteration indicates a dysfunction in TJ and a decrease in the expression of the ZO-1 molecule. Similar patterns have been observed in BCBM [124,144]. For a summary of key contributors and their influence on the BBB- and TJ-associated proteins, refer to Table 1.

By critically analyzing and understanding the roles of TJ and AJ proteins in BM, we can develop potential therapeutic strategies. Restoring and enhancing the expression of claudin-1 or stabilizing claudin-5 could potentially inhibit cancer cell invasion and metastasis [12]. Additionally, utilizing Focused Ultrasound (FUS) therapy and Nanoparticles (NPs) to manipulate these structural proteins could improve drug delivery, a topic we will discuss in the next section.

## 9. Innovative Preservation: Enhancing Metastasis Therapy with Focused Ultrasound and Nanoparticles

### 9.1. Focused Ultrasound Therapy

**Figure 5:**
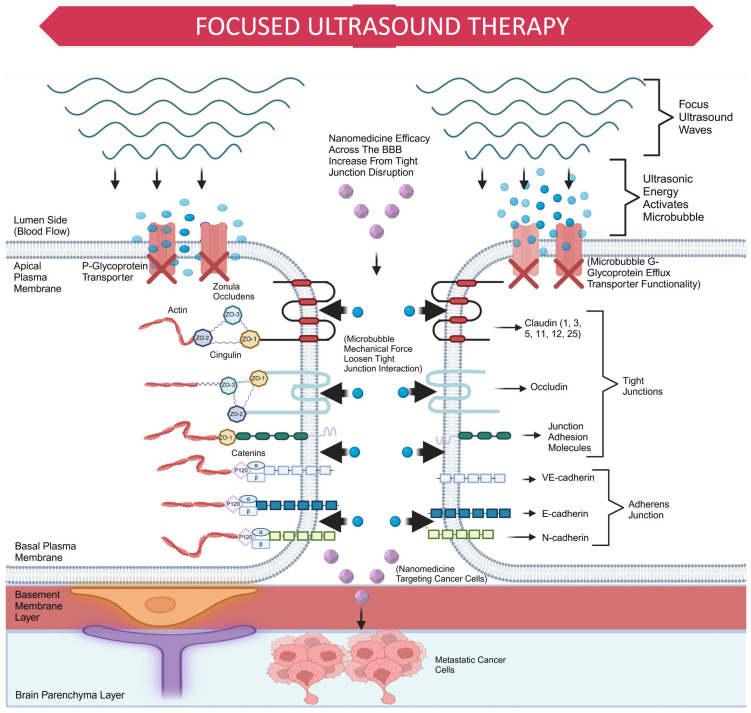
Schematic illustration of FUS therapy and its effects on the BBB. The diagram shows how FUS waves activate microbubbles, which exert mechanical forces on the TJ proteins of BMECs. This mechanical action disrupts TJ interactions, specifically affecting proteins such as Claudin (1, 3, 5, 11, 12, and 25), occludin, and JAMs. Additionally, the process inhibits efflux transporters, such as P-glycoprotein, increasing the permeability of the BBB. This enhanced permeability allows for improved drug delivery to metastatic cancer cells in the brain parenchyma layer via nanomedicine.

FUS therapy temporarily increases the permeability of the BBB using ultrasound radiation and microbubbles. Low-intensity ultrasonic energy activates microbubbles to expand and contract, creating mechanical forces that target BMECs. This process loosens TJ proteins and inhibits the activity of efflux transporters like P-gp, resulting in an increased retention of small molecules in the brain delivered by nanomedicines (see Figure 5) [170].

However, the transient disruption of the BBB induced by FUS may lead to several potential adverse effects, including bleeding, brain damage, obnubilation (clouding of consciousness), and brain inflammation. These side effects require close monitoring and highlight the importance of balancing therapeutic benefits with safety concerns. To achieve maximum efficiency for brain tumor treatment and minimize potential side effects, it is essential that we carefully optimize the parameters of FUS, such as frequency and power [171]. This optimization can be achieved by utilizing Magnetic Resonance (MR)-guided Focused Ultrasound (MRgFUS) therapy, which provides real-time imaging to ensure precise targeting and reduce the risk of complications [171].

MRgFUS therapy has shown great potential in clinical treatment, where the MRI guides the process, providing real-time feedback, monitors temperature changes, and ensures the procedure’s safety [172]. A clinical study utilized MRgFUS to administer the monoclonal antibody trastuzumab to four patients with Her2-positive BM. The treatment proved safe and effective, with increased drug delivery to the MRgFUS-targeted lesions compared to the nontargeted lesions [173]. Despite its promising results, it is crucial to continue evaluating the long-term safety and efficacy of FUS therapy to address any potential limitations and ensure the best outcomes for patients. Standardizing protocols and parameters for BBB disruption is essential for robust clinical translation. By optimizing MRgFUS parameters to minimize potential side effects while maximizing drug delivery efficiency, and exploring the potential of combining FUS with other treatment modalities, such as immunotherapy and gene therapy, can lead to a significant enhancement in therapeutic outcomes.

### 9.2. Nanoparticle Drug Delivery

NPs are being studied as a promising method for delivering drugs across the BBB with unparalleled precision in monitoring treatment and delivery. A range of NP types, such as polymeric, liposomes, dendrimers, quantum dots, and nano-gels, are available [174]. Polymeric NPs, made from polymers such as polyethylene glycol (PEG) or polylactide-co-glycolide (PLGA), encapsulate drugs to enhance bioavailability and therapeutic efficacy [174]. These NPs can be engineered for a controlled and sustained drug release, targeted therapy to specific tissues or cells, and improving the solubility of poorly water-soluble drugs [171,174]. Utilizing biodegradable polymers in their engineering minimizes long-term accumulation and potential toxicity while ensuring biocompatibility to avoid adverse reactions. Polymeric NPs have applications in drug delivery, gene therapy, diagnostics, and imaging, such as delivering siRNA for gene silencing in cancer therapy [174].

Liposome NPs, engineered from a phospholipid bilayer, can encapsulate both hydrophilic and lipophilic drugs, making them versatile for drug delivery [174]. They offer a reduced risk of adverse effects due to their biocompatibility and advancements in targeted therapy, allowing liposome NPs to bind to specific cells and enhance drug delivery precision, exemplified by Caelyx for recurrent malignant glioma [171,174]. Liposomes also have stable characteristics, improved membrane fluidity, and the ability to evade the immune system, enabling effective drug delivery [174].

Dendrimers are tree-like macromolecules with a high degree of surface functionality and versatility [174]. Their ability to encapsulate drugs enhances solubility and targeting capabilities, as seen with poly(amidoamine) (PAMAM) dendrimers encapsulating anticancer drugs like doxorubicin [171,174]. Dendrimers are useful in drug delivery, gene therapy, diagnostics, and imaging due to their biocompatibility and reduced risk of adverse reactions. However, they face challenges such as high toxicity [171,174].

NP utilizes diverse transport mechanisms to navigate the BBB, including paracellular transport through TJ proteins and adsorptive-mediated transcytosis. Additionally, receptor-mediated transcytosis is utilized, which involves transferrin and low-density lipoprotein, along with transporter-mediated transcytosis, including glutathione transporters, and L-type amino acid transporters [171].

Despite NP being viewed as a potential tool for drug delivery, it does have several limitations and adverse effects. It has been shown that inter-endothelial openings in tumor blood vessels are infrequent and do not constitute the primary mode of NP transport into solid tumors. Moreover, the passive extravasation mechanism employed by NP was regularly observed to account for only a small portion of NP tumor accumulation, with further analysis revealing that 97% of NP infiltrate tumors through an active process via endothelium cells [175].

Neurotoxicity is a significant concern regarding NPs. NPs have been shown to exert toxic effects on the BBB, disrupting its integrity and leading to the formation of reactive oxygen species, which can damage cells and cause necrosis or apoptosis, resulting in neuronal loss due to their interactions with the negatively charged cell membranes [174]. High concentrations of NPs can also be toxic to the BBB, inducing microglia-mediated inflammatory responses and further compromising BBB integrity. Additionally, there is a risk of NPs affecting non-target cells or tissues, leading to unintended side effects. Ensuring that NPs remain at the target site and are not quickly cleared from the body also remains a challenge [174].

Strategies being analyzed for NP drug delivery include the use of lipid polymeric NPs modified with peptides FD7 or CCD to deliver Afatinib, a drug commonly used to treat NSCLC BM. FD7 is known for its ability to modulate TJ proteins, particularly claudins-5 and ZO-1, enhancing ion permeability and disrupting TJ proteins on the BBB [176]. CCD peptide, a cyclic derivative from the extracellular 1 domain of human E-cadherin, modulates E-cadherin-mediated cell–cell adhesion and inhibits the AJ of vascular endothelial cells forming the BBB. The results demonstrated that these modified NPs released the drug gradually without damaging brain cells, improving its efficacy against cancer cells. The peptides FD7 and CCD effectively interfered with TJ proteins, thereby reducing resistance in the BBB and increasing its permeability [176]. Micelles have also shown potential for targeting BCBM by incorporating paclitaxel (PTX) and lapatinib (LPTN) as nano-vehicles. The efficacy of these micelles was enhanced through Angiopep-2 modification, resulting in the development of modified Ang-MIC-PTX/LP micelles. An in vitro model showed that these modified micelles crossed the BBB and accumulated in metastatic cells with positive results [177].

A promising technique for combating BCBM involves a ‘trojan horse’ strategy. By encapsulating doxorubicin in an NP cloaked with the cell membrane of a cancer cell that specifically targets the brain, the drug can be precisely delivered to the cancer cells within the brain. This resulted in the suppression of cancer cell proliferation and tumor size reduction [178]. Emerging evidence has also revealed the utilization of NP and near-infrared light to enhance cancer treatment. These NPs are designed to respond to the tumor’s acidic environment and release a potent stress granule inhibitor, which sensitizes cancer cells to photothermal therapy. This innovative approach employs a dual-action mechanism that not only triggers an immune response but also modulates TAMs. Preliminary findings suggest this approach holds significant potential for metastasis therapy [179].

NPs hold significant therapeutic potential for delivering nanomedicine across the BBB with unmatched precision. However, their clinical application must address challenges such as neurotoxicity, adverse effects on BBB integrity, and unintended side effects on non-target cells. Efforts should focus on enhancing biocompatibility, improving targeting and penetration efficiency through advanced mechanisms, integrating NPs with other therapeutic modalities, conducting extensive in vivo studies for clinical translation, and establishing standardized protocols for production and regulatory approval. By addressing these areas, the full potential of NPs in drug delivery across the BBB can be realized, leading to improved therapeutic outcome.

## 10. Future Direction and Conclusions

The BBB plays a pivotal role in the development and progression of BM. As tumors advance, key BBB components shift from protective guardians to facilitators of BM, secreting pro-tumorigenic factors. Future research must prioritize elucidating the mechanisms underlying these transitions, particularly the microglia polarization into TAM-MG and the role of microglial-derived exosomes in BM progression. Developing therapies to modulate microglial polarization and converting M2 microglia to the cytotoxic M1 phenotype holds significant promise for therapeutic intervention.

In astrocytes, the transition from defensive roles to supporting tumor growth, as well as their involvement in extracellular-vesicle-mediated communication and immune evasion, warrants further investigation. Disrupting astrocyte–tumor cell interactions, targeting key signaling pathways such as STAT3, CHI3L1, and PPAR-gamma, and developing microRNA-based therapies are promising strategies for inhibiting astrocyte-mediated tumor progression.

Understanding the factors and gene expression changes in pericytes during tumor interactions is critical, as these cells exhibit potential pro-tumorigenic functions. Targeting pericyte–tumor interactions, including the inhibition of PDGF-BB signaling and IGF2 pathways, could counteract their growth-promoting effects on BM cells. Additionally, further studies are needed to explore the role of oligodendrocytes, neuron–tumor interactions such as neurotransmitter signaling and reelin resurgence, and cannabinoid receptors in controlling BM growth. Research gaps related to changes in BMECs and glycocalyx dynamics are crucial for improving BM treatment. Understanding how the glycocalyx layer influences tumor cell adhesion and migration and the role of HS levels in cancer cell attachment is essential.

Efflux transporters, such as P-gp and MRPs, and SLC transporters play significant roles in multidrug resistance and BM progression. Research must focus on elucidating the mechanisms underlying efflux transporter overexpression and developing strategies to inhibit their function, improve drug delivery, and overcome resistance. Combination therapies incorporating efflux transporter inhibitors and personalized treatment plans based on transporter expression profiles could enhance treatment efficacy. Addressing the research gaps in understanding the disruptions and expression patterns of TJ and AJ proteins is also vital. Methodological advancements should focus on developing therapies to restore and enhance TJ protein expression and utilizing FUS therapy and NPs to manipulate these structural proteins. Utilizing advanced single-cell sequencing techniques such as CITE-seq offer unparalleled insights into cellular interactions, disease mechanisms, and therapeutic targets. These tools should be leveraged to explore changes in these BBB cellular components for developing immuno-therapy and personalized treatment strategies.

Innovative therapeutic approaches, such as FUS therapy and NPs, hold promise for improving drug delivery and efficacy. However, these methods still face limitations. Further research and technical advancements in FUS are crucial for enhancing its speed and precision and facilitate combination therapies. For NPs, it is essential that we investigate the mechanisms of the NP–tumor interface and conduct a detailed analysis of the surface proteins on NPs in conjunction with tumor endothelial cells. Innovative strategies should aim to influence the tumor endothelium to boost the trans-endothelial transport of NPs. It is vital that we clarify the function of various tumor vessels and distinct trans-endothelial routes concerning the extravasation of NPs of diverse sizes, shapes, and surface chemistries. Additionally, understanding the role of cell types, such as immune cells, in creating temporary permeability, or utilizing FUS, should be prioritized.

## Figures and Tables

**Table 1:** A Non-Exhaustive Overview of Key Contributors Influencing the BBB and TJ-Associated Proteins.

**Primary** **Metastases Tumor**	**Factors/Protein**	**Mechanism of Action**	**References**
Lung Cancer	Adenosine A2A receptor	Activation leads to an upregulation of TJ proteins (claudin-5, occludin, and Z0-1)	[145]
	ALCAM	Upregulation increases tumor cell adhesion to BMEC and dissemination	[146]
	miR-143–3p	Altered gene expression increases TJ proteins	[147]
	Visfatin	Induces upregulation of CCL2, causing downregulation of claudin-5, Z0-1, and Z0-2	[148]
	PLGF	Disassembly of TJ proteins	[149]
	MMP-2 and -9	ECM degradation; targets TJ proteins	[150,151]
Breast Cancer	Angiopoietin-2	Impairs TJ protein (ZO-1 and claudin-5)	[152]
	Cathepsin S	Proteolytic cleavage of JAM-B	[153]
	S1P3	Astrocytic secretion of IL-6 and CCL2, resulting in BMEC adhesion relaxation	[154]
	SEMA4D	Promotion of angiogenesis and cancer cell invasiveness	[155]
	CXCL13 and CXCL1	Increases BBB permeability	[156]
	VEGF	Disruption of endothelial monolayers	[157]
	SP	Decreased TJ proteins, ZO-1, and claudin-5; and induced BBB Damage	[158]
	lncRNA GS1-600G8.5	Silencing of GS1-600G8.5 significantly abrogated the BBB destructive effect of exosomes	[159]
	miR-181c	Degradation of PDPK1, resulting in BBB breakdown	[160]
	miR-105	Targets TJ protein Z0-1	[161]
Melanoma Cancer	Serine Protease	Disruption of TJ proteins	[162]
	uPAR	Degradation of ECM	[163,164]
	TGF-β	Decreased in claudin-5 and TEER	[165]
	Heparanase	Degradation and fragmentation of ECM	[166,167]
	MMP-9	Targets TJ proteins and ECM degradation	[168]
	VLA-4	Intercalation and destabilization of BMEC and TJ proteins	[169]

## Abbreviations

BM	Brain Metastasis
CNS	Central Nervous System
ME	Microenvironment
BMECs	Brain Microvascular Endothelial Cells
TJ	Tight Junction
AJ	Adherens Junction
NVU	Neurovascular unit
JAM	Junction Adhesion Molecules
ZO	Zonula Occludens
BME	Basement Membrane
ECM	Extracellular matrix
GFAP	Glial Fibrillary Acidic Protein
GDNF	Glial Cell Line-Derived Neurotrophic Factor
OPCs	Oligodendrocyte Precursor Cells
GAG	Glycosaminoglycan
FGFR	Fibroblast Growth Factor Receptor
PGs	Proteoglycans
HS	Heparan Sulfate
CS	Chondroitin Sulfate
ABC	ATP-Binding Cassette
SLC	Solute Carriers
P-gp	P-glycoprotein
MRP	Multidrug Resistance-Associated Protein
TMD	Transmembrane Domain
NBD	Nucleotide-Binding Domain
TGF-β	Transforming Growth Factor-β
TAM-MG	Tumor-Associated Macrophage
NT	Neurotrophin
NSCLC	Non-Small Cell Lung Cancer
MBM	Melanoma Brain Metastasis
BCBM	Breast Cancer Brain Metastasis
LCBM	Lung Cancer Brain Metastasis
dLGG	1,2-di-O-α-linolenoyl-3-O-β-galactopyranosyl-sn-glycerol
CSF-1R	Colony Stimulating Factor 1 Receptor
EVs	Extracellular Vesicles
CCL2	Chemokine Ligand 2
CCR2	Chemokine Receptor Type 2
CVC	Cenicriviroc
CX43	Connexin 43
pSTAT3	Phosphorylated STAT3
CHI3L1	Chitinase 3-like-1
BCSCs	Breast Cancer Stem Cells
SCLC	Small Cell Lung Cancer
PDGFB	Platelet-Derived Growth Factor-B
PFT	Pericyte-to-Fibroblast Transition
IGF2	Insulin-like Growth Factor 2
PPP	Picropodophyllin
PSMA	Prostate-Specific Membrane Antigen
CB1R	Cannabinoid CB1 Receptor
BTB	Blood-Tumor Barrier
BAT	Brain Area Surrounding The Tumor
CTCs	Circulating Tumor Cells
FUS	Focus Ultrasound
NP	Nanoparticles
MRgFUS	Magnetic Resonance (MR)-guided Focused Ultrasound
PEG	Polyethylene glycol
PLGA	Polylactide-co-glycolide
PTX	Paclitaxel
LPTN	Lapatinib
